# Towards prevention of post-traumatic osteoarthritis: report from an international expert working group on considerations for the design and conduct of interventional studies following acute knee injury

**DOI:** 10.1016/j.joca.2018.08.001

**Published:** 2019-01

**Authors:** F.E. Watt, N. Corp, S.R. Kingsbury, R. Frobell, M. Englund, D.T. Felson, M. Levesque, S. Majumdar, C. Wilson, D.J. Beard, L.S. Lohmander, V.B. Kraus, F. Roemer, P.G. Conaghan, D.J. Mason, Arthritis Research UK Osteoarthritis and Crystal Disease Clinical Study Group Expert Working Group, J. Adams, M. Blank, M. Batt, P. Biggs, M. Busse-Morris, K. Button, J. Calder, J. Cook, C. Edwards, E. Fisheleva, D.F. Hamilton, H. Harrison, C. Holt, M. Jones, R. Jones, S. Kluzek, T. Knight, G. Nuki, S. Parekh, G. Peat, C. Pothet, T. Rainer, N. Robinson, L. Sawle, T. Vincent, A. Williams, E. Wise, W. Zhang, S. Bierma-Zeinstra

**Affiliations:** §§§ Faculty of Health Sciences, University of Southampton, UK; ‖‖‖ Nottingham University Hospitals and Arthritis Research UK Centre for Sport, Exercise and Osteoarthritis, University of Nottingham, UK; ¶¶¶ School of Engineering, Cardiff University, UK; ### School of Medicine, Cardiff University, UK; †††† School of Healthcare Sciences, Cardiff University, UK; ‡‡‡‡ Fortius Clinic, London, UK; §§§§ Nuffield Department of Orthopaedics, Rheumatology and Musculoskeletal Sciences, University of Oxford, UK; ‖‖‖‖ Faculty of Medicine, University of Southampton, UK; ¶¶¶¶ Medicines Research Centre, Glaxo-SmithKline, UK; #### School of Clinical Sciences, University of Edinburgh, UK; ††††† University of Salford, UK; ‡‡‡‡‡ School of Molecular, Genetic and Population Health Sciences, University of Edinburgh, UK; §§§§§ Great Ormond Street Institute of Child Health, University College London, UK; ‖‖‖‖‖ Arthritis Research UK Primary Care Centre, Research Institute for Primary Care & Health Sciences, Keele University, UK; ¶¶¶¶¶ Health and Social Care/Allied Health Services, London South Bank University, UK; ##### Arthritis Research UK Centre for Osteoarthritis Pathogenesis, Kennedy Institute of Rheumatology, Nuffield Department of Orthopaedics, Rheumatology and Musculoskeletal Sciences, University of Oxford, UK; †††††† Encompass Healthcare, Washington, Tyne and Wear, UK; ‡‡‡‡‡‡ Department of General Practice, Department of Orthopedics, Erasmus MC – University Medical Centre, Rotterdam, The Netherlands; † Arthritis Research UK Centre for Osteoarthritis Pathogenesis, Kennedy Institute of Rheumatology, Nuffield Department of Orthopaedics, Rheumatology and Musculoskeletal Sciences, University of Oxford, Roosevelt Drive, Oxford, OX3 7FY, United Kingdom; ‡ Arthritis Research UK Primary Care Centre, Institute for Primary Care & Health Sciences, Keele University, Keele, UK; § Leeds Institute of Rheumatic and Musculoskeletal Medicine, University of Leeds & NIHR Leeds Biomedical Research Centre, Leeds, UK; ‖ Lund University, Faculty of Medicine, Department of Clinical Sciences Lund, Orthopaedics, Lund, Sweden; ¶ Clinical Epidemiology Research & Training Unit, Boston University School of Medicine, Boston, MA, USA; # NIHR Biomedical Research Centre, University of Manchester, Manchester, UK; †† Immunology Development, Abbvie Bioresearch Center, Worcester, MA, USA; ‡‡ Musculoskeletal Quantitative Imaging Research Group, Department of Radiology & Biomedical Imaging, University of California San Francisco, San Francisco, USA; §§ Dept of Trauma and Orthopaedics, University Health Board, Cardiff, UK; ‖‖ Surgical Intervention Trials Unit (SITU), Nuffield Department of Orthopaedics, Rheumatology and Musculokeletal Sciences, University of Oxford, Oxford, UK; ¶¶ Duke Molecular Physiology Institute and Division of Rheumatology, Duke University School of Medicine, Durham, USA; ## Department of Radiology, University of Erlangen-Nuremberg, Erlangen, Germany; ††† Department of Radiology, Boston University School of Medicine, Boston, MA, USA; ‡‡‡ Arthritis Research UK Biomechanics and Bioengineering Centre, School of Biosciences, Cardiff University, Cardiff, UK

**Keywords:** Osteoarthritis, Injury, Outcome, Clinical trial, Knee, Considerations

## Abstract

**Objective:**

There are few guidelines for clinical trials of interventions for prevention of post-traumatic osteoarthritis (PTOA), reflecting challenges in this area. An international multi-disciplinary expert group including patients was convened to generate points to consider for the design and conduct of interventional studies following acute knee injury.

**Design:**

An evidence review on acute knee injury interventional studies to prevent PTOA was presented to the group, alongside overviews of challenges in this area, including potential targets, biomarkers and imaging. Working groups considered pre-identified key areas: eligibility criteria and outcomes, biomarkers, injury definition and intervention timing including multi-modality interventions. Consensus agreement within the group on points to consider was generated and is reported here after iterative review by all contributors.

**Results:**

The evidence review identified 37 studies. Study duration and outcomes varied widely and 70% examined surgical interventions. Considerations were grouped into three areas: justification of inclusion criteria including the classification of injury and participant age (as people over 35 may have pre-existing OA); careful consideration in the selection and timing of outcomes or biomarkers; definition of the intervention(s)/comparator(s) and the appropriate time-window for intervention (considerations may be particular to intervention type). Areas for further research included demonstrating the utility of patient-reported outcomes, biomarkers and imaging outcomes from ancillary/cohort studies in this area, and development of surrogate clinical trial endpoints that shorten the duration of clinical trials and are acceptable to regulatory agencies.

**Conclusions:**

These considerations represent the first international consensus on the conduct of interventional studies following acute knee joint trauma.

## Introduction

Osteoarthritis (OA) pathologically represents a continuum from risk exposure, to molecular changes and structural changes with associated pain, which for some people progresses to the need for joint replacement. Detection and treatment of those at high risk of OA could enable effective interventions before any major structural damage has occurred or before pain becomes chronic, that is at a pre-radiographic or even pre-symptomatic stage. Such intervention would be comparable to current early management of diabetes, cardiovascular disease, osteoporosis or pre-rheumatoid arthritis.

Joint injury remains one of the biggest risk factors for OA. In Sweden, approximately 80/100,000 people per year experience anterior cruciate ligament (ACL) rupture; in the U.S. there are 252,000 ACL injuries per year^1^, ^2^. 50% of people with significant knee joint injuries such as ACL rupture and/or acute meniscal tear subsequently develop symptomatic radiographic OA within 10 years, so-called post-traumatic OA (PTOA)^3^; at least 33% with acute ACL rupture will have magnetic resonance imaging (MRI)-defined whole joint OA after 5 years^4^. PTOA is thought to comprise around 12% of all OA, although its incidence appears to be increasing^5^, ^6^. However, there are no specific guidelines for clinical trials which seek to measure the effect of interventions for prevention of OA after an injury^7^, ^8^. There are a number of challenges in study design specific to this area, especially the potentially long study duration needed. As such, regulatory considerations include the identification of surrogate outcomes for PTOA studies and the creation of a new indication: OA prevention. This has led to significant uncertainty for regulatory agencies and drug developers, and has restrained investments by the pharmaceutical industry.

An international expert working group was therefore convened with the following aims: to review the literature on existing interventional studies close to the time of knee injury; give an overview of key areas in the field relevant to future interventional studies; define considerations for the conduct and design of trials aimed at prevention of OA; and to highlight knowledge gaps by developing research recommendations in this area.

## Methods

The considerations process was facilitated by the Osteoarthritis and Crystal Diseases Clinical Studies Group of Arthritis Research UK (UK's largest arthritis charity), which was established to develop consensus research priorities and nurture methodologically robust clinical trials.

Whilst preventing joint injury is an intervention to prevent PTOA^7^, our focus was on interventions *after* knee joint trauma. We conducted an evidence review, then consensus process developing considerations and a research agenda. Though the evidence review summarized the use of outcome measures including patient reported outcome measures (PROMs), no recommendations for specific outcome measures were planned.

### Evidence review

An evidence review was conducted to identify experimental, interventional studies following acute knee injury with specific reference to post-traumatic knee OA. Systematic searches were conducted across five databases (Cochrane Library; EMBASE; MEDLINE; CINAHLPlus; AMED) from inception to August 2016. The search strategy was designed in OVID-Medline using text words and subject headings (MeSH), combining terms for knee injury, osteoarthritis and clinical trials or systematic reviews (Supplementary Table 1).

All references were imported into Endnote where duplicates were removed. Screening and study detail extraction was by NC, verified by three others (FW, DM, PC). Study inclusion criteria were as follows: population clearly stated within 6 months of acute knee injury (any setting); interventional study (any intervention, including surgical, pharmacological, non-pharmacological) with any comparator (including active, placebo, sham or no intervention); OA or a surrogate outcome measure; reported randomized controlled trials (RCTs), non-randomized controlled trials or systematic reviews. Study exclusion criteria included: ‘acute’ injury not clearly separated from ‘chronic’, or from other joint disease; non-English-language articles; letters, comments or editorials. Observational studies of interventions in this area were not included in our evidence search or considerations, as they were felt to be prone to bias and not representative of our main focus which related to experimental studies.

### Consensus group

A group of 32 stakeholders, including physiotherapists, orthopaedic surgeons, rheumatologists, sports and exercise medicine physicians, primary care physicians, radiologists, laboratory scientists, statisticians, clinical trialists, engineers, pharmaceutical company experts and four patient representatives (2 who had a previous knee joint injury) comprised the consensus group. After the evidence review results were circulated, the group convened at a face-to-face meeting. The evidence review, which included a summary on the use of PROMs, was presented and overviews of literature-identified key areas were given by invited experts: challenges around studies in this area (Lohmander), molecular biomarkers (Kraus) and imaging (Roemer). Specific case study examples of potential interventional targets and challenges were presented (Mason, Kraus). Three working groups with facilitators and reporters were convened to consider: A: Eligibility criteria and choice of outcomes, B: The use of biomarkers (including soluble biomarkers and imaging) as potential stratifiers or outcomes, and C: Definition of the injury, the timing of intervention, and considerations for multi-modality interventions. Written notes were compiled, presented by each group's reporter to all stakeholders and agreement on items and additional overarching points to consider were generated during a final discussion session, chaired by PC, with written statements agreed by all (facilitated by FW). The meeting was taped and transcribed; any uncertainties were addressed from the transcript. Subsequently, the document and then manuscript was reviewed by all contributors through an iterative online process.

## Results

### Evidence review

The initial search identified 2476 citations (MEDLINE, *n* = 532; EMBASE, *n* = 863; CINAHLPlus, *n* = 489; AMED, *n* = 60; Cochrane Library, *n* = 532). 945 duplicates were removed. Screening of the remaining 1531 abstracts yielded 43 eligible studies. Seven systematic reviews identified a further 15 reported trials. From these 58 papers (including 11 conference abstracts), 37 unique studies were included. Details of each study are summarized in Supplementary Table 2. The majority of studies involved ACL injury (*n* = 20; 54%), patellar dislocation (*n* = 8; 22%) or tibial plateau fracture (*n* = 7; 19%), with the remaining two studies including any ‘acute knee injury’.

Table I summarizes the basic study details grouped according to type of injury. All but two studies were RCTs (*n* = 35; 95%). Of 16 studies reporting power calculations, 15 met or exceeded the sample size required (Supplementary Table 2). Study duration varied widely, approximately equally distributed across 0–1 years, >1–5 years and >5 years. Most studies (70%) compared a surgical intervention against either another surgical or non-surgical/non-pharmacological (henceforth referred to as ‘other’) intervention. Comparisons of post-operative rehabilitation interventions, pharmacological studies (the only studies to use a placebo arm) and all other interventions each accounted for ∼8% of all studies (Table I).

**Table I:** Overview of basic study details, categorized according to type of knee injury

	ACL	Patellar Dislocation	Tibial plateau fracture	Other	Total
**Number of studies [papers if different]**	20 [40 papers incl. 11 conf. abstracts]	8 [9 papers, incl. 1 abstract only]	7 incl. 2 abstract only	2 studies	37 [58 papers incl. 11 conf. abstracts & three abstracts only]
**RCT/nRCT**	RCT: 20 incl. 1 protocol and 1 pilot	RCT: 7 nRCT: 1	RCT: 6 incl. 1 protocol nRCT: 1	RCT: 2 incl. 1 pilot	RCT: 35 incl. 2 pilots, 2 protocol; nRCT: 2
**Sample size at randomization**					
Missing	1				1
<20	2			1	3
20-50	5	5	4		14
>50–100	7	2	2		11
>100–200	5	1	1	1	8
**Power calculation**					
*a priori*	9	5	2		16
*post hoc*	2			1	3
None	8	2	3	1	14
Unclear	1	1	2		4
**Study adequately powered** (based on sample size)	9 of 9	4 of 5	2 of 2		15 of 16
**Study duration**					
Missing		1	1		2
<3 months	1			1	2
3–6 month	3				3
>6 months–1 year	3		3	1	7
>1–2 years	4	3			7
>2–5 years	3		2		5
>5–10 years	2	3			5
>10 years	4	1	1		6
**Primary outcome measure(s) clearly defined**	9 + 1 used for sample size calculation	5	2 + 1 used for sample size calculation	1	17 + 2 based on sample size calculation
**Type of interventions**					
Surgical vs Surgical	10		7		17
Surgical vs Other	1	8			9
Other vs Other	3				3
Pharmacological vs Pharmacological	2 (all placebo)			1 (placebo)	3: all placebo controlled
Post-op Rehab vs Post-op Rehab	2			1	3
Post-op Pharma vs Post-op Pharma	1				1
Post-op Pharma vs No intervention	1				1

ACL = Anterior cruciate ligament.

RCT = Randomized controlled trial.

nRCT = non-randomized controlled trial.

An overview of inclusion and exclusion criteria for all available full-text papers (*n* = 32) is shown in Supplementary Table 3. Most studies (88%) had clearly defined eligibility criteria. Sixty percent provided a specific age range, spanning 13–50 years old. Sex was a specified criterion in only three studies, one of which excluded females. Elite professional sports activity and pregnancy were exclusions in 20% of studies.

Pre-existing conditions or other concomitant injuries excluded patients in 80% of studies. For example, previous index (and sometimes contralateral) knee injury and/or surgery were exclusions in >60% of studies and the presence of OA was an exclusion criterion in 25% of studies (Supplementary Tables 3 and 4).

One-hundred-and-forty-seven outcome measures were identified (Table II), including physical examination outcomes (*n* = 30), patient-reported outcomes (*n* = 26) of which the Knee Injury and Osteoarthritis Outcome Score (KOOS) was most frequently used^5^, imaging outcomes (*n* = 43), biomarkers (*n* = 39) and other (*n* = 9) (Supplementary Tables 5–9 respectively). Primary outcome measures were identified by only 19 studies (Table I, Table II). Ten different OA outcomes included nine imaging structural measures and one surrogate measure, KOOS (Table II, Supplementary Tables 6 and 7). Only five studies (of ACL rupture subjects) used molecular biomarkers as outcome measures (Supplementary Table 8).

**Table II:** Summary of outcome measures

Outcome measure category (n*)	Primary outcomes	Osteoarthritis and surrogate OA outcomes
Physical examination *n* = 30	1. Laxity (*n* = 4) 2. Patellofemoral stability (*n* = 2) 3. Limb symmetry indices (*n* = 1) 4. Torque (*n* = 1) 5. Muscle electrical activity (*n* = 1) 6. Functional – hop test (*n* = 1)	
Patient reported *n* = 26	1. Knee injury and Osteoarthritis Outcome Score (KOOS) (*n* = 5) 2. Hospital for Special Surgery (HSS) knee score (*n* = 2) 3. International Knee Documentation Committee (IKDC) Subjective Knee Form (*n* = 2) 4. Kujala score (*n* = 2) 5. The Knee Self-Efficacy Scale (K-SES) (*n* = 1) 6. The Physical Activity Scale (*n* = 1) 7. Tegner activity score (*n* = 1) 8. Multidimensional Health Locus of Control (*n* = 1)	1. Knee injury and Osteoarthritis Outcome Score (KOOS) (*n* = 1)
Imaging *n* = 43	1. Radiographic: Kellgren–Lawrence classification (*n* = 1) 2. CT: Quality of reduction (*n* = 1) 3. MRI: Morphologic measures of articulating bone curvature (femur, tibia & trochlea) (*n* = 1) 4. MRI: Cartilage thickness of femorotibial medial compartment (*n* = 1) 5. MRI: Anterior Cruciate Ligament Osteoarthritis Score (ACLOS) (*n* = 1)	1. Radiographic: Study specified criteria incl. joint space narrowing, osteophyte grade, subchondral sclerosis and sharpening of tibial spines (*n* = 4) 2. Radiographic: Kellgren–Lawrence classification (*n* = 4) 3. Radiographic: Ahlbäck classification (*n* = 2) 4. Radiographic: modified OARSI grading scale for OA (*n* = 1) 5. Radiographic: Medial joint space width (*n* = 1) 6. Radiographic: Ahlbäck & Fairbank composite scale (*n* = 1) 7. MRI: Whole Organ Magnetic Resonance Imaging Score (WORMS) (*n* = 1) 8. MRI: Anterior Cruciate Ligament Osteoarthritis Score (ACLOAS) (*n* = 1) 9. qMRI: Early matrix changes typical of arthritis (*n* = 1)
Biomarkers (*n* = 39)	1. GAG/proteoglycan marker: ARGS-aggrecan (*n* = 1)	
Other (*n* = 9)	2. Safety, tolerability & adverse effects (*n* = 1)	
**TOTAL *n*** = **147**	**Total** = **21 primary outcomes**	**Total** = **10 measures**

*where n = total number of studies with such measures.

### Summary of key area discussions

#### Molecular pathogenesis and biomarkers of the injury response

Recently there has been an increase in our understanding of the molecular pathogenesis of PTOA. Observations from both humans and animal models reveal that diverse signalling pathways (involving inflammation, apoptosis and cell senescence) are activated by injury^10^, ^9^. This activation is associated with subsequent bone remodelling, cartilage matrix damage and synovial inflammation^11^, ^12^. Synovial fluid at the time of joint injury shows marked increases in pro-inflammatory cytokines (e.g., IL-6 is 1000-fold up-regulated) and within 2 weeks shows evidence of matrix catabolism of both aggrecan and type II collagen^13^, ^14^, ^15^. The response appears to differ between individuals, and is represented by a tissue inflammatory response, primarily detectable in the synovial fluid^13^, ^14^, ^16^. Following injury, a variety of factors may encourage joint homeostasis and resolution (including normal physiological loading), or progression to post-traumatic OA (including excessive loading or further injury). Further injury or surgery would appear to prolong the inflammatory response to trauma^17^. There may be an ‘early therapeutic window’ following joint injury during which inflammatory response genes are up-regulated and matrix degradation is initiated which could be targeted by intervention^18^. The optimal and/or latest times at which degradation could be halted or reversed are currently unknown.

#### Animal models of knee injury

Much work on OA pathogenesis has been accomplished in animal models, which exploit the association between joint injury and OA, using trauma or surgically-induced injury to predictably induce disease: they are therefore particularly suited to testing early interventions in this setting. Findings from murine models such as those involving destabilization of the medial meniscus appear to translate to human studies of ACL rupture or meniscal tear^14^. The effects of suppressing certain key pathways in these models have been described in knockout mice^19^. Despite this, very few interventions have been tested at the time of injury, in rodents or in man, as opposed to established OA, which could account for some of the failure of translation of OA therapeutics to date.

However, there may be some molecular differences as well as some practical challenges in the testing of intra-articular agents in small animals and in the extrapolation of optimal timing of an intervention from rodent to man**.**

#### Examples of potential pharmacological targets

Glutamate concentrations are increased in synovial fluid of arthritic joints in humans and animals, activating glutamate receptors on neurones and synoviocytes to induce pain and cause release of IL-6^20^, ^21^. Intra-articular inhibition of α-amino-3-hydroxy-5-methyl-4-isoxazolepropionic acid (AMPA) and kainate glutamate receptors at the time of injury or induction of arthritis in rodent models alleviates pain, inflammation and joint degeneration^22^, ^23^. IL-1 causes cartilage degradation *in vitro* and is upregulated in synovial fluid following joint injury^24^, ^25^. Blockade of this pathway (with IL1-receptor antagonist (IL1RA)) reduced inflammation and degeneration in a mouse model of arthritis^26^. IL-1 or AMPA/kainate receptors represent potential therapeutic targets for preventing later disease, as their inhibition at the time of injury in models of post-traumatic OA reduced disease. IL1RA is the first therapeutic agent to be tested in human pilot studies at the time of knee injury for this indication^27^. A further example is a small RCT testing steroid injection within 4 days of ACL tear, where the collagen degradation biomarker CTX-II was significantly reduced in synovial fluid in the steroid-treated arms^15^. Since AMPA/kainate receptor antagonists, IL1RA and steroids are already used in man, re-purposing of existing agents is a real possibility.

#### Imaging in acute injuries

Imaging-based change following knee injury reflects the initial trauma but also the responses to subsequent changed dynamic knee loading after destabilizing injuries^28^. The majority of studies include X-ray and MRI cartilage outcomes, both semi-quantitative and quantitative. Although there are a few high-quality longitudinal imaging studies after ACL rupture, more studies are needed. It is possible to define early OA on either X-ray or MRI, and evidence indicates that MRI changes alone can act as an endpoint^29^. Depending on the target, non-cartilage MR outcomes, either bone-based, such as bone marrow lesions (BMLs) or synovitis-effusion, may be appropriate. Compositional measures using MRI, positron emission tomography (PET) or computed tomography (CT) remain investigational. Composite metric sequences including T_1ρ_ and T_2_ have been associated with the PROM KOOS, pain after ACL reconstruction and with synovial fluid biomarkers at the time of surgery^30^, ^31^, ^32^. Change in these compositional measures may reflect differences in surgical factors after ACL reconstruction and the pre-injury joint structure^33^.

Consistent changes in cartilage thickness occur after ACL rupture: two cartilage regions quickly increase in thickness over time, whilst other areas decrease^34^. Within 3 months of ACL injury, there are marked changes in knee bone curvature^35^. Patellofemoral joint (PFJ) OA appears more prevalent in cohort studies, particularly relating to ACL rupture/reconstruction; however, the PFJ is not always examined by X-ray.

Structural changes generally develop slowly, and traumatic and degenerative changes must be clearly separated, although may appear similar (as in the case of BMLs). Common OA assessment semi-quantitative instruments are only partially applicable in this setting: Whole Organ Magnetic Resonance Imaging Score (WORMS), BLOKS and MOAKS do not differentiate between traumatic and degenerative joint changes, and do not include assessment of post-surgical graft integrity^36^. Anterior Cruciate Ligament Osteoarthritis Score (ACLOAS) is a new tool which addresses some of these issues including clear differentiation of traumatic from degenerative BMLs, extent of baseline traumatic osteochondral damage and assessment of the graft^37^.

#### Imaging biomarkers predicting OA

A systematic review in this area reported that meniscal lesions, meniscectomy, BMLs, time from injury and altered biomechanics all are associated with cartilage loss over time after ACL rupture^38^. Greater cartilage damage at baseline is associated with worse clinical outcome (although this could represent pre-existing OA)^39^, ^40^, ^41^. Presence of cortical depression fractures is associated with a worse International Knee Documentation Committee (IKDC) score at 1 year^42^. MRI-detected inflammation markers (effusion-synovitis/Hoffa-synovitis) at 2 years after ACL rupture were associated with OA development at 5 years^43^. Effusion, or presence of BMLs at 1 year, or meniscal tears at any stage were found to be associated with radiological OA at 2 years^39^. Early bone curvature change is predictive of cartilage loss at 5 years and accentuated by the presence of meniscal injury^35^.

### Points to consider

These are summarized under overarching considerations and three main areas: eligibility criteria, outcome measures and definition and timing of interventions and comparators in these studies.

#### Overarching considerations

Key overarching considerations are included in Table III. It was emphasized that a better understanding of disease pathogenesis was important. The appropriate time-window, role and effects of a proposed intervention on underlying processes such as inflammation, mechanical loading and subsequent bone or cartilage change need to be elucidated. Some findings may usefully be translated from animal models; however, it was also noted that there may be important differences between the response to acute knee trauma and a discrete surgically-induced isolated injury to ACL or meniscus. It was agreed that the considerations highlighted in this paper should be reviewed periodically as more data become available, with a maximum of 3 years before the next revision.

**Table III:** Recommendations for points to consider: overarching considerations

Consideration	Recommendation
1. General	□ Considerations should be relevant to the design of all forms of interventional study following joint injury, unless otherwise stated □ CONSORT or STROBE criteria should be adopted in the design and reporting of any interventional or cohort study in this area □ Patients and the public should be involved throughout the process of study design and delivery
2. Regulatory	□ Current and future regulatory considerations and requirements in this area should be considered in design of future studies □ The community should work closely with regulatory bodies to establish evidence and precedent for outcomes and design of interventional trials □ Responder criteria, the number needed to treat for benefit (NNT), and cost-effectiveness should be measured
3. Feasibility	□ Feasibility, patient burden and cost considerations of, for example, type of imaging, or intervening near to the injury should be carefully weighed against the scientific/therapeutic benefits of the proposed approach □ For any given study, a balance should be found between scientific rigour in design and pragmatic considerations regarding recruitment and generalizability to clinical practice
4. Specific targets	□ Some of the considerations around study design (including eligibility criteria, outcomes and time-window of intervention) may be different, depending on the nature of the intervention □ There may be particular biomarker(s) which are specific and sensitive for a particular intervention
5. Stratification	□ The assessment of personal or individualized risk was noted to be important □ Novel molecular or imaging biomarkers might be used in the future as stratifiers at the point of entry to the study, or as intermediate (surrogate) outcomes, but none are validated for these purposes currently □ Effective stratification of an individual's personal risk of post-traumatic OA is not yet possible based on current knowledge

#### Eligibility criteria

Eligibility criteria should be clearly defined and should identify specific groups with a modifiable process following their injury in which to test the intervention (Table IV).

**Table IV:** Recommendations for points to consider: eligibility criteria

Consideration	Recommendation
1. Definition of acute knee injury	□ The extent and characteristics of acute structural joint damage should be fully classified by magnetic resonance imaging □ Subgroups/types of injury for inclusion such as ACL and/or meniscal tear should be carefully defined □ Different types of injury may be associated with different biomechanical outcomes and responsiveness to any given intervention, so the target population needs to be carefully defined □ In the case of meniscal tears, the individual's age, history of a clear injurious episode, plus MR appearances are all important in identifying traumatic tears (and excluding degenerative lesions from these studies) □ Caution should be exercised in the inclusion of extreme phenotypes, for example those with isolated ACL tears or very extensive injuries
2. Time since injury	□ Establishing an appropriate therapeutic time-window will be relevant for each new target/intervention □ Certain interventions targeting the early response to injury may benefit from being tested within days of injury, or up to a maximum of 4–6 weeks from injury
3. Age	□ Upper age limit should be carefully considered; an upper age limit of 35 was proposed □ Challenges were highlighted around intervening in paediatric populations who lack capacity to give informed consent or who have immature growth plates
4. Demographics	□ People of both sexes should be included □ Studies may include, but should not be restricted, to professional athletes
5. Proposed exclusions	□ Other existing causes of joint pathology ○ inflammatory arthritis or pre-existing established osteoarthritis ○ other disorders of bone, current or past ○ previous substantial injury or surgery of index knee (particularly where there would be an associated markedly increased risk of PTOA) ○ other concomitant body injury or surgery (in some circumstances as may confound biomarkers) □ Pregnancy or breast-feeding □ Heavy use of alcohol, or recreational drug use □ Morbid obesity

##### Definition of injury

Examples of well-defined groups based on MRI to be included would be ACL tear combined with other injuries such as traumatic meniscal tear (although different outcomes are probably associated with medial or lateral tears)^44^, or chondral damage/cortical depression fracture^42^. Degenerative meniscal lesions should be considered part of early OA and *not* included in acute post-trauma studies^45^. 55% of patients sustain simultaneous injuries to both ACL and meniscus^46^; the ubiquitous biological response to joint tissues injury supports broader inclusion of injury sub-types. Combined ligament injuries or fractures should not necessarily be excluded but considered as a separate ‘extreme’ phenotype, as they may be at substantially increased OA risk, which may or may not be reversible.

##### Time since injury

Some interventions may be most effective if exerting their effect as soon as possible after the early biological changes after injury. The appropriate time window for any intervention after injury needs to be carefully justified, according to it's nature.

##### Age

Those less than 30 years are more likely to have purely traumatic meniscal lesions; those over age 35 could be at risk of pre-existing OA/degenerative meniscal lesions.

##### Demographics

Elite athletes are more likely to have past/repeated injuries but may have different responses to injury compared to non-elite individuals. As elite athletes are at high risk of OA, they still represent a relevant subgroup for investigation.

##### Exclusions

Previous substantial knee injury or surgical procedure to the index knee may confound results and should be considered as a possible exclusion. BMI should be documented: excessive obesity has independent effects on disease risk, joint loading and inflammation.

#### Outcome measures

Key considerations are shown in Table V.

**Table V:** Recommendations for points to consider: outcome measures

Consideration	Recommendation
1. General	□ Measures of symptoms and structure are both important and should be recorded □ The primary outcome measure(s) are likely to be required after 1–2 years after intervention but should relate to the study question □ Short, medium and long term outcomes should be collected □ Frequent outcomes should be considered in the first year, particularly for efficacy and biomarker-related questions
2. Patient reported outcome measures (PROMs)	□ PROMs which have been validated within appropriate populations and which examine pain, function, performance and quality of life were recommended □ The choice of tool should depend on its extent of validation and reliability as well as feasibility including cost □ Early assessment of the cost effectiveness of any given intervention, or interventions should be considered
3. Imaging	□ Imaging should be used a) to categorize and phenotype, and b) as an important outcome measure □ MRI and X-ray are both important outcome measures, but MRI may have increased sensitivity at earlier times after injury □ The patello-femoral joint and tibio-femoral joints should both be included in imaging assessments □ An index/signal knee should be defined (given that the opposite side may subsequently be affected) □ The contralateral knee may be a useful imaging control or comparator for the index/signal knee □ The index/signal knee, and ideally both knees, should be imaged at 0 (baseline), 12 months and 24 months for structural changes after intervention; inclusion of a later time point, such as 5 years was also recommended □ Morphology and change in all joint tissues should be captured, using validated semi-quantitative and/or quantitative measures □ Compositional assessment at 6 months for cartilage (MRI) or bone changes (MRI, PET, CT) is more experimental but should be considered in addition to structural assessments
4. Molecular biomarkers	□ No specific biomarker(s) can be recommended for routine use in interventional studies ○ Biomarkers cannot yet act as independent surrogate endpoints for early OA diagnosis ○ Biomarkers have not been validated for aiding selection of patients for interventional studies □ Molecular biomarkers should be considered as exploratory outcome measures in interventional studies ○ Choice(s) will depend on the target and outcomes under study □ Bio-samples (including synovial fluid, in addition to serum/plasma and urine) should be collected in all future studies where possible ○ Serum and urine should be collected at all available time points ○ Sampling should include DNA storage where appropriate consent is given ○ Synovial fluid can be accessed at the time of surgery or clinical aspiration, or at the time of drug delivery into the index/ signal knee ○ Timing and method of sample collection must be consistent and standardized across all studied patients
5. Functional outcomes	□ Stability of the knee and muscle strength are important to patients, and potentially important outcome measures □ Symptoms of instability may have value in addition to examination-based measures of mechanical instability/laxity □ Other potential functional biomarkers include kinematics, hop or stair climbing tests and muscle co-contraction testing

##### PROMs

In addition to the collection of longer-term PROMs, repeated, multiple early measures will allow examination of potential earlier surrogate endpoints in the future.

##### Imaging

Baseline and longitudinal evaluation should differentiate pre-existing degenerative from acute traumatic structural joint damage. The contralateral knee may subsequently be affected, therefore differentiating index from control knee is important. Considerations around type of imaging and its frequency include evidence of specific outcome performance metrics, feasibility and cost. Where trials are multi-center, MRI protocols need to be carefully designed (for example, compositional imaging may be challenging in a multi-center setting, and magnet strength should be considered in the context of ACL reconstruction and metal artefact). Selection of imaging biomarker (semi-quantitative or quantitative) requires understanding of the validity, reliability and responsiveness of each measure. MRI techniques that assess early cartilage changes may be useful. Measures of synovial or fat pad inflammation may be important for anti-inflammatory therapeutics and MRI techniques that quantify synovitis may be considered. Early changes in 3D bone shape seen after injury which predict subsequent OA warrant further study as a potential surrogate endpoint.

##### Molecular biomarkers

These were noted to be under development as stratifiers and as outcome measures: none were yet sufficiently evidence-based to act as independent surrogate measures as either an early OA diagnostic, prognostic or patient selection aid for interventional studies. Irrespective of target, to accelerate therapeutic advances, it is important that bio-samples be collected in all cohorts and clinical trials where possible. DNA storage would allow the international community to work collaboratively to identify novel genetic predictors of outcome.

Synovial fluid was highlighted as a potentially important biosample, showing biologically important molecular changes after injury and after intervention; synovial fluid molecular changes are likely to have increased utility compared to serum^13^, ^14^, ^15^. Contralateral aspiration of synovial fluid was controversial, as the contralateral knee is not always a good control and it is difficult to aspirate normal joints. It is important that non-surgical studies access synovial fluid to avoid bias towards surgical intervention studies. In some cohorts, serum/plasma/urine may be available prior to the injury (e.g., participants in a biobank or military cohorts): measuring change within an individual was noted as analytically powerful. Regarding biomarker choice, the most qualified biomarkers to date, e.g., CTX-II, could be included if cartilage matrix catabolism is a target; synovial inflammation or bone biomarkers, or specific cytokine measurements may be relevant depending on target^27^.

##### Functional outcomes

Symptoms of instability could be more reliable than any examination-based measures. However, their sensitivity to change compared with existing measures such as pain should be evaluated further^47^.

#### Definition and timing of intervention and comparator

The choice of timing of the intervention will depend on the nature and mode of its action and intended effects, as well as the measured outcome. An optimal ‘therapeutic window’ should be carefully defined for any intervention (Table VI), see also Eligibility Criteria: ‘Time Since Injury’. It may be that identification of high risk phenotypes is possible by imaging or molecular biomarkers at defined times after the injury.

**Table VI:** Recommendations for points to consider: definition and timing of intervention and comparators

Consideration	Recommendation
1. General	□ Optimal time-window for administration of any given intervention should be validated and clearly defined □ Assumptions should be avoided; different proposed time-windows for intervention should be tested head to head in feasibility studies if necessary, to ensure patient acceptability, recruitment and likely translation in to clinical care
2. Comparators	□ A comparator and/or placebo or sham arm should always be used where possible ○ Choice will depend on whether study is efficacy or pragmatic ○ Patients should be randomized to intervention or comparator arms ○ Assessment of acceptability of sham treatments, particularly when invasive, is paramount when considering design and feasibility □ Double blind protocols should be used where possible □ While double-blinding is not always possible, blinded observer/assessor almost always is
3. Multimodality intervention	□ Multi-modality interventions may be particularly suited to this area ○ Such studies are very challenging to design and deliver and require expert input ○ Choice of each component ideally requires *a priori* evidence of effect □ The interaction of different interventions is an important consideration in this area, given that multi-modal intervention is common in clinical practice.

Types of intervention are highly varied; where multi-modality interventions are used, these should be carefully defined, and controlled. Drugs could be given systemically or intra-articularly, as single or multiple doses, dependent on agent and duration of treatment, safety considerations and acceptability.

A comparator and/or placebo or sham arm should be used, because of the known substantial placebo effect in OA studies^48^. The comparator will often be standard or usual care, rather than no treatment and requires careful definition. Randomization and placebo control are important principles not only for pharmacological interventions, but also for device and surgical studies, where a large placebo effect would be anticipated and which is not otherwise controlled^49^.

There are a number of practical considerations for successful recruitment, randomisation strategies, the standardisation of the intervention (particularly if surgical) and allocation concealment in these types of studies, particularly when they are multi-site^18^. This should be carefully considered during study design and a number of existing OARSI recommendations in trials of prevention of joint injury and of established OA are highly relevant here^50^, ^51^, ^7^, ^8^.

### Research recommendations

The particular challenges and questions highlighted as needing further research are included in Table VII.

**Table VII:** Research recommendations

Consideration	Recommendation
1. General	□ To best define populations to be included in studies, further work is needed to understand relative risk of OA in different injury types, identifying ○ Injuries which are easily defined and categorized and are at high risk of OA ○ Injuries for which this risk is likely to be reversible ○ Injuries particularly suited to different types of intervention □ Further work to enable prediction/stratification of individual risk of future OA at the time of injury, using clinical factors imaging and/or molecular biomarker profiling is needed ○ These predictors should be examined alone but also in combination □ Further work on defining the appropriate time-window for intervention after joint injury is needed ○ This may differ depending on the nature of the proposed intervention and the population studied
2. Pre-clinical studies	□ The analogous nature of animal models of post-traumatic OA was highlighted, and the potential to therefore support translational interventional studies in human □ Animal models or experimental medicine studies in human should be used to define the likely best delivery of an intervention, its optimal time-window and initial pharmacokinetics, to support future clinical trials
3. Preparation for translation	□ Patient and public involvement should be sought, particularly around areas of assessing risk of disease, risk of harm, risk of overtreatment and acceptability of different types of proposed interventions □ Feasibility studies are encouraged to address questions specific to an intervention, acceptability to patients, and refine best outcomes. Findings should be published, to enable shared knowledge.
4. Outcomes	□ Better evidence for the modality and timing of early imaging as an outcome measure is needed □ Evidence to support the use of surrogate outcomes of efficacy is needed: clinical/PROMs-based, imaging-based or biomarker-based, linking these early outcomes to later disease risk □ Evidence for the recommendation of one or more PROMs with the best utility in this area should be sought □ Longer observational/cohort/clinical trials should be designed to collect information on: ○ natural history of joint trauma and outcomes ○ utility of molecular biomarkers ○ relationship between PROMs, biomarkers and imaging outcomes ○ relationship between early outcomes (at 1 or 2 years) and later outcomes at 5–10 years □ Close liaison with industry and with regulatory authorities on the areas of outcomes research and clinical need is advised to achieve an indication in this area

Patient representatives highlighted concerns for the potential for over-diagnosis or overtreatment in the absence of risk stratification, and further Patient and Public Involvement is encouraged in this area now, and as the field develops.

Further evidence is needed for which outcomes should be used in this setting, and what measurement(s) (whether a molecular or imaging biomarker or PROM) might act as an acceptable surrogate short term outcome for future OA (given that 5–10 year interventional trials are not feasible). Although these current considerations address interventional studies, the consensus group acknowledged that ancillary/cohort studies which establish associations between PROMs, biomarkers and imaging outcomes could address key knowledge gaps to provide evidence for future trials. The design of these studies should be carefully considered and outcomes appropriately powered, but they may include more exploratory outcomes. Sensitive, specific early measures which might shorten studies should be sought.

The Consensus group noted that animal studies can inform human studies, and such programs were justifiable to facilitate early translation of targets to humans.

## Discussion

Our review of the literature has highlighted a lack of conformity in design of interventional studies in this area. Evidence from the review and expert consensus has been synthesised in producing these first international considerations on the design and conduct of interventional studies aiming at prevention of OA following acute knee injury. Critical knowledge gaps limiting such trials have been highlighted, and summarised as research recommendations. These considerations are intended to underpin future guidelines as this field evolves. Collaborative working on cohort and feasibility studies is needed to provide better evidence for interventional study design.

Studies need to include those patients who are at the highest risk, but whose risk is modifiable by the proposed intervention. There was an awareness of the identity of extreme phenotypes, such as combined ligament injuries, which may fall outside these criteria. As in OA, predictive risk modelling is needed for knee trauma^52^. A better understanding of underlying disease mechanisms from both animal and human studies is needed. Understanding how related mechanisms such as inflammation and mechanical loading of the joint after trauma contribute to either resolution or progression to OA was deemed essential for the development of new interventions.

The feasibility and acceptability of testing interventions in an acute setting can be challenging. Informed consent for sham or placebo treatments at the time of knee injury needs careful review by patients, healthcare providers and trialists. Sham-controlled trials including surgical trials are often needed to provide the best possible level of evidence^49^. Recent consensus in classification of early knee OA will facilitate such trials^53^. Alternative surrogate outcome measures need to be developed to shorten trial duration and improve the likelihood of drugs being developed by industry. MRI costs are relatively high, but may be justified by allowing researchers to examine earlier outcomes. Whilst X-ray follow-up may appear more feasible, it's use as a lone imaging modality must be adequately powered.

There are some limitations to the approach used. The literature review was performed to provide evidence for discussions, rather than as a stand-alone piece of work; it was clear after the initial search that areas of interest, such as pharmacological interventions, were not well represented in the current literature, and limitations of generalizability to all types of interventions should therefore be borne in mind. A critical appraisal of the studies was not performed as it was not felt necessary for the requirements of this review, which was pragmatic in nature. Given the relatively low number of RCTs identified in this area, non-randomized controlled trials as well as RCTs were included where identified. Not all opinions might be equally represented from this type of approach. However, a wide range of stakeholders and groups were involved, including patients. Effort was made to ensure diversity; pre-appointed facilitators and reporters with note-keeping and voice recording of sessions ensured a transparent and consistent process. More detailed discussions on considerations of recruitment/randomization/allocation concealment strategies were beyond our scope^54^.

In summary, these initial considerations provide a starting point for further work in this area. These points are intended to be complimentary to, and should be considered alongside, OARSI Clinical Trials Recommendations on prevention of joint injury, the design, analysis and reporting of OA RCTs and clinical requirements for development of therapeutics in OA^50^, ^51^, ^55^, ^7^. The regulatory considerations for a new indication of preventing symptoms or OA structural change following joint injury are unique. Engagement with both regulators and the pharmaceutical industry is essential if the area is to progress and overcome current hurdles. Although such trial designs may be challenging, in order to develop new therapeutics with the aim of patient benefit, the consensus was that progress in this area is both possible and urgently required.

## Author contributions

All authors made substantial contributions to all three of sections (1), (2) and (3) below:

(1) acquisition of data, or analysis and interpretation of data.
(2) drafting the article or revising it critically for important intellectual content.
(3) final approval of the version to be submitted.

DJM, FEW and PGC in addition conceived and designed the work, and take collective responsibility for data integrity as a whole.

## Competing interest statement

FEW – none.

NC – none.

SK – none.

RF – none.

ME – none.

DF –none.

ML – employee of Abbvie pharmaceutical company.

SM – none.

CW – none.

DB – none.

SL – none.

VBK – none.

FWR – Shareholder Boston Imaging Core Lab. (BICL), LLC., a company providing radiologic image assessment services to academia and the pharmaceutical industry.

DJM – co-inventor on a patent related to the use of glutamate receptor antagonists to prevent osteoarthritis (WO2015001349).

PGC – none.

## Role of the funding source

The meeting was funded by Arthritis Research UK as part of the Clinical Studies Group for Osteoarthritis and Crystal Diseases programme. In addition, the Arthritis Research UK Centre for Osteoarthritis Pathogenesis (20205 and 21621) and Arthritis Research UK Biomechanics and Bioengineering Centre (20781) gave support via their joint organization of the meeting. FEW and PGC are also part of the Arthritis Research UK Centre for Sports, Exercise and Osteoarthritis (21595). No representation from Arthritis Research UK was present at the meeting. No specific funding was received from any other funding bodies in the public, commercial or not-for-profit sectors to carry out the work described in this manuscript.

PGC and SK are supported in part through the NIHR Leeds Biomedical Research Centre, and FEW in part by the NIHR Oxford Biomedical Research Centre and the Kennedy Trust for Rheumatology Research. The views expressed are those of the authors and not necessarily those of the NHS, the NIHR or the Department of Health.
