# Supplementary material for: Towards prevention of post-traumatic osteoarthritis: report from an international expert working group on considerations for the design and conduct of interventional studies following acute knee injury
Source: Osteoarthritis Cartilage. 2019 Jan;27(1):23–33. doi: 10.1016/j.joca.2018.08.001 (PMC6323612; doi:10.1016/j.joca.2018.08.001)
Supplement: Multimedia component 1 [file mmc1.docx]

# Supplementary Tables

**Supplementary Table 1. Search strategy**

**MEDLINE OvidSP(ran 30.08.16)**- Epub Ahead of Print, In-Process & Other Non-Indexed Citations, Ovid MEDLINE(R)Daily and Ovid MEDLINE(R)1946 to Present

Clinical trials filter – Cochrane Highly Sensitive Search Strategy for identifying RCTs in MEDLINE: sensitivity- and precision-maximising version (2008 revision): Ovid format

| 1 | Knee/ |
| --- | --- |
| 2 | exp Knee Joint/ |
| 3 | (knee or knees).ti,ab,kw. |
| 4 | patella/ |
| 5 | patell*.ti,ab,kw. |
| 6 | Anterior Cruciate Ligament/ |
| 7 | Posterior Cruciate Ligament/ |
| 8 | medial collateral ligament, knee/ |
| 9 | (femorotibial or femoro tibial).ti,ab,kw. |
| 10 | (tibiofemoral or tibio femoral).ti,ab,kw. |
| 11 | or/1-10 |
| 12 | exp "Wounds and Injuries"/ |
| 13 | hemarthrosis/ |
| 14 | (haemarthrosis or hemarthrosis).ti,ab,kw. |
| 15 | trauma*.ti,ab,kw. |
| 16 | injur*.ti,ab,kw. |
| 17 | rupture*.ti,ab,kw. |
| 18 | (tear* or torn).ti,ab,kw. |
| 19 | fracture*.ti,ab,kw. |
| 20 | dislocation*.ti,ab,kw. |
| 21 | or/12-18 |
| 22 | 11 and 21 |
| 23 | exp Knee Injuries/ |
| 24 | ((posttrauma* or post trauma*) adj5 effusion).ti,ab,kw. |
| 25 | (cruciate* adj5 (tear* or torn or rupture* or injur* or trauma*)).ti,ab,kw. |
| 26 | ((ACL or PCL) adj5 (tear* or torn or rupture* or injur* or trauma*)).ti,ab,kw. |
| 27 | (menisc* adj5 (tear* or torn or rupture* or injur* or trauma*)).ti,ab,kw. |
| 28 | (collateral* adj5 (tear* or torn or rupture* or injur* or trauma*)).ti,ab,kw. |
| 29 | ((MCL or LCL) adj5 (tear* or torn or rupture* or injur* or trauma*)).ti,ab,kw. |
| 30 | ((combined or multiple) adj5 ligament* adj5 (tear* or torn or rupture* or injur* or trauma*)).ti,ab,kw. |
| 31 | ((multiligament* or multi ligament*) adj5 (tear* or torn or rupture* or injur* or trauma*)).ti,ab,kw. |
| 32 | ((posterolateral or postero lateral) adj5 (tear* or torn or rupture* or injur* or trauma*)).ti,ab,kw. |
| 33 | ((posteromedial or postero medial) adj5 (tear* or torn or rupture* or injur* or trauma*)).ti,ab,kw. |
| 34 | ((MCL or LCL) adj5 (tear* or torn or rupture* or injur* or trauma*)).ti,ab,kw. |
| 35 | ((PMC or PLC) adj5 (tear* or torn or rupture* or injur* or trauma*)).ti,ab,kw. |
| 36 | (tibia* adj3 (eminence* or tuberosity or plateau) adj5 fracture*).ti,ab,kw. |
| 37 | ((femoral or femur) adj3 condyle* adj5 fracture*).ti,ab,kw. |
| 38 | or/23-37 |
| 39 | 22 or 38 |
| 40 | Osteoarthritis/ |
| 41 | Osteoarthritis, Knee/ |
| 42 | OA.ti,ab,kw. |
| 43 | osteoarthr*.ti,ab,kw. |
| 44 | or/40-43 |
| 45 | randomized controlled trial.pt. |
| 46 | controlled clinical trial.pt. |
| 47 | randomized.ab. |
| 48 | placebo.ab. |
| 49 | clinical trials as topic.sh. |
| 50 | trial.ti. |
| 51 | or/45-50 |
| 52 | (systematic adj2 review).ti. |
| 53 | (meta analysis or metaanalysis).ti. |
| 54 | 52 or 53 |
| 55 | 51 or 54 |
| 56 | exp animals/ not humans/ |
| 57 | 55 not 56 |
| 58 | 39 and 44 and 57 |

## Supplementary Table 2: Basic study details. Abbreviations: *n* – sample size at randomization; ITT – intention to treat; Non-RCT – non-randomized comparative/controlled trial; IA – Intraarticular. ACL -Anterior Cruciate Ligament 1° Outcome: SS – included as primary outcome based on use in sample size calculation, note – yes includes ‘main or primary outcome[s]; - unspecified as conference abstract/abstract only.

## ACL injuries

|  | **Study (papers)** | **Journal** | **Design** | **Country** | **Setting** | **Condition** | **Time from injury to intervention/ enrolment** | **Intervention1** | **Intervention2** | ***n***  **[excl after]** | **Study Time** | **1**°  **Out-come** | ***a priori* power calc.** |
| --- | --- | --- | --- | --- | --- | --- | --- | --- | --- | --- | --- | --- | --- |
| A1 | Akelman, Fadale, Hulstyn, *et al.* (2016). | Am J Sports Med | RCT ; ITT analysis; 60 matched healthy controls | USA | Clinics, 3 surgeons | Isolated unilateral ACL injuries | ≤ 12 months before 1st office visit | **Surgical:** Low tension (bone-patellar tendon-bone or 4-stranded hamstring autograft) | **Surgical:** High tension (bone-patellar tendon-bone or 4-stranded hamstring autograft) | 90 | 7y | SS | Yes  (*n=45 a group*) |
| A1a: Fleming, Fadale, Hulstyn, *et al.* (2013). Am J Sports Med (3 year results) | | | | | | | | | | | | | |
| A2 | Barenius, Ponzer, Shalabi, et al. (2014). | Am J Sports Med | RCT; 2 centres | Sweden | Knee surgeons well experienced in both methods | Traumatic ACL injury with instability | ≥ 2 months. Early (<5 mos) vs later reconstructions. | **Surgical:** Arthroscopic reconstruction with an ipsilateral bone–patellar tendon–bone graft | **Surgical:** Arthroscopic reconstruction with an ipsilateral quadrupled semi-tendinosus graft | 180 [16] | 14y | Yes | No |
| *A2a: Barenius, Nordlander, Ponzer, et al. (2010). Am J Sports Med (8 year results)*  A2b: Eriksson, Anderberg, Hamberg, *et al.* (2001). J Bone Joint Surg Br (2 year results) | | | | | | | | | | | | | |
| A3 | Beynnon, Johnson, Naud, *et al.* (2011). | Am J Sports Med | RCT; ITT analysis | USA | University-affiliated private clinic, 2 orthopaedic surgeons. | ACLrepair (bone–patellar tendon–bone graft) | Index knee injury to surgery: range = 13-134 days | **Post-op rehab:** Accelerated rehabilitation (19 week) | **Post-op rehab:** Non-accelerated rehabilitation (32 week) | 42 | 2y | Yes | Yes  (*n*=15 per group) |
| A4 | Beynnon, Uh, Johnson, *et al.*  (2005). | Am J Sports Med | RCT | USA | University Sports Medicine Center,.2 surgeons | ACL tear | Diagnosed within a 2-week time interval of the injury | **Post-op rehab:** Accelerated rehabilitation (19 week) | **Post-op rehab:** Non-accelerated rehabilitation (32 week) | 25 | 2y | Yes | Yes  (*n*=10 per group) |
| A5 | Cicuttini, Wang, Marks, *et al*. [Conference abstract] | Osteoarthritis Cartilage | RCT  (2:1 allocation); Phase 1b/2a. |  |  | Unilateral ACL injury requiring reconstruction within 6 months | 4-6 weeks following reconstruction carried out within 6 months of original injury | **Post-op pharma:** Single IA injection of 75 million allogeneic mesenchymal precursor cells+ hyaluronan | **Post-op pharma:** Single IA injection of hyaluronan | 17 | 2y | Yes | Unclear |
| A6 | Drogset, Grontvedt, Robak, *et al.* (2006). | J Bone Joint Surg Am | RCT | Norway | 3 university hospitals | Acute ACL rupture | Operation within 10 days of injury | **Surgery1:** Acute primary repair.  **Surgery2:** Acute repair + autologous bone-patellar tendon-bone graft | **Surgery3:** Acute repair + synthetic ligament-augmentation device | 150 | 16y | No | No |
| A7 | Karimi-Mobarakeh, Mardani-Kivi, Mortazavi, *et al.* (2015). | Knee Surg Sports Traumatol Arthrosc | RCT | Iran |  | ACL tear | ≥ 1.5 months prior to the study [Mean 2.7 +/- 1.9 and 2.8+/-1.6 months] | **Surgical:** Semitendinosus autograft reconstruction | **Surgical:** Semitendinosus + gracilis reconstruction | 129 | 1y | No | Yes  (*n*=49 per group) |
| A8a | **KANON trial**  Frobell, Roos, Roos, *et al.* (2013). | BMJ | RCT, multicentre. Extended follow up | Sweden | Orthopaedic department of 2 hospitals; 4 knee surgeons, procedure used was surgeon’s preference | Acute ACL tear in previously uninjured knee | Within 4 weeks of injury | **Surgical:** Rehabilitation + early ACL reconstruction (ACLR, ≤10 weeks of injury, patella-tendon or hamstrings-tendon procedure) | **Surgical:** Rehabilitation + delayed ACL repair if needed | 141 [20] | 5y | Yes | Yes  (*n*=120 total) |
| A8b Frobell, Roos, Roos, *et al.* (2010). NEJM (2 years)  A8c Frobell, Roos, Roos, *et al.* (2008). Osteoarthritis Cartilage  A8d Ericsson, Roos, & Frobell (2013). BMJ (ancillary study; ‘as treated’ approach; 87 of original 121 patients)  A8e Roemer, Frobell, Lohmander, Niu, & Guermazi (2014). Osteoarthritis Cartilage (subgroup of 20 patients)  A8f Roessler, Andersen, Lohmander, & Roos (2015). Scandinavian Journal of Medicine & Science in Sports (secondary analysis; 2 years)  A8g Filbay, Frobell, Lohmander, & Roos (2015). Osteoarthritis Cartilage (Exploratory analysis: early and delayed ACLR as one group (n=89)) [Conference abstract]  A8h Bowes, Lohmander, Wolstenholme, *et al.*. (2013). Osteoarthritis Cartilage (5 years) [Conference abstract]  A8i Eckstein, Wirth, Lohmander, *et al.* (2014). Osteoarthritis Cartilage [Conference abstract]  A8j Frobell, Lohmander, Onnerfjord, *et al.* (2014). Osteoarthritis Cartilage [Conference abstract]  A8k Hunter, Lohmander, Makovey, *et al.* (2013). [Conference abstract] Osteoarthritis Cartilage  A8l Larsson, Frobell, Lohmander, & Struglics (2016). Osteoarthritis Cartilage (analysed "as treated") [Conference abstract]  A8m Larsson, Hansson, Frobell, Lohmander, & Struglics (2013). Osteoarthritis Cartilage (21 knee-healthy individuals used as reference) [Conference abstract]  A8n Struglics, Larsson, Kumahashi, Frobell, & Lohmander (2015). Osteoarthritis Cartilage (5 years) [Conference abstract]  A8o Wirth, Eckstein, Hudelmaier, Lohmander, & Frobell. (2013). Osteoarthritis Cartilage (analysed "as treated", 5 years) [Conference abstract] | | | | | | | | | | | | | |
| A9 | Koken, Akan, Kaya, *et al.* (2014). | Eur Orthop Traumatol | RCT; single blind | Turkey |  | ACL deficiency | Average 7 weeks (range 3–24 weeks) | **Surgical:** Arthroscopic reconstruction with anatomic single bundle | **Surgical:** Arthroscopic reconstruction with double bundles | 67 | 1y | No | No |
| A10 | Kraus, Birmingham, Stabler, *et al.* (2012). | Osteoarthritis Cartilage | RCT pilot study; proof-of-concept pilot trial | USA | Duke Sports Medical clinic | Acute ACL tear confirmed by MRI | Treated within 30 days of injury | **Pharma:** Single intra-articular injection of IL-1Ra (Anakinra 150mg) | **Pharma:** Single intra-articular injection, saline placebo (equal volume 1ml) | 11 | 28d | Yes | No (*post hoc*) |
| A10a Catterall, Stabler, Flannery, *et al.* (2010). Arthritis Research & Therapy  A10b Catterall, Stabler, & Kraus (2009). Arthritis Research & Therapy [Conference abstract] | | | | | | | | | | | | | |
| A11 | Lawrence, Birmingham, & Toth (2011). | Clin Orthop Relat Res | **PROTOCOL** RCT; Phase II | USA |  | Acute isolated ACL tears |  | **Pharma:** Intra-articular IL-1 inhibitor | **Pharma:** Placebo | - | 2y | No | No |
| A12 | Magnussen, Pottkotter, Stasi, *et al.* (2016). | The Journal of Knee Surgery | RCT | USA | Three sports medicine fellowship-trained orthopedic surgeons | Acute isolated ACL tear |  | **Post-op pharma:** Femoral nerve block (1 shot) following hamstring autograft ACL reconstruction | **No intervention:** Hamstring autograft ACL reconstruction only | 30 | 6m | Yes | Yes  (*n*=24 total) |
| A13 | Meunier, Odensten, & Good (2007). | Scand J Med Sci Sports | RCT | Sweden | Emergency ward, University Hospital Linkoping. | Acute, complete ACL tear |  | **Surgical:** ACL repair | **Non-surgical:** Conservative treatment | 100 | 15y | No | No |
| A14 | Sun, Zhang, Wang *et al.* (2012). | Knee Surg Sports Traumatol Arthrosc | RCT | China | Single arthroscopic surgeon | Primary unilateral reconstructions of the ACL. | 0.5-5.2 months | **Surgical:**  Irradiated hamstring tendon allograft | **Surgical:** Non-irradiated hamstring tendon allograft | 78 | 43m | No | No |
| A15 | Sun, Tian, Zhang, *et al.* (2009). | Arthroscopy | RCT | China | Single arthroscopic surgeon. | ACL tear | 0.5-5.5 months | **Surgical:** Bone-patellar tendon-bone autograft | **Surgical:** Bone-patellar tendon-bone allograft | 172  [5] | 6y | No | No |
| A16 | Swirtun, Jansson, & Renstrom (2005). | Clin J Sport Med | RCT | Sweden | Sports Medicine Clinics | Clinically verified acute ACL tear | <5 weeks post-injury | **Non-surgical:** Functional bracing from baseline to 12 weeks postinjury | **Non-surgical:** No bracing | 95  [53] | 6m | Yes | No |
| A17 | Tagesson, Oberg, Good, *et al.* (2008). | Am J Sports Med | RCT | Sweden | Orthopaedic department. | Unilateral ACL tear | ≤14 weeks (range 20-96 days) | **Non-surgical:** Rehabilitation incl. **c**losed kinetic chain quadriceps exercises | **Non-surgical:**  Rehabilitation incl. open kinetic chain quadriceps exercises | 49 | 16w | No | Yes  (*n*=19 per group) |
| *A18* | *Taylor, DeBerardino, Nelson, et al. (2009).* | *Am J Sports Med* | *RCT, stratified by surgeon* | *USA* | *Keller Army Hospital, NY; 4 surgeons* | *Complete ACL tear* | *Acute i.e. <3 m (n=47); Chronic (n=17)* | ***Surgical:*** *4-strand hamstring autograft ACL reconstruction* | ***Surgical:*** *Patellar tendon autograft ACL reconstruction* | *64* | *4y* | No | Yes  (*n*=60 total) |
| A19 | Thomee, Wahrborg, Borjesson, *et al.* (2010) [Abstract only] | J Sport Rehabil | RCT |  | 8 physio-therapists | ACL tears |  | **Non-surgical:** Standard rehabilitation with physiotherapists given patients' self-efficacy scores | **Non-surgical:** Standard rehabilitation. with physiotherapists not given patients' self-efficacy scores | 40 | 1y | Yes | Yes  (*n*=20 per group) |
| A20 | Webster, Feller, Hartnett, *et al.* (2016). | Am J Sports Med | RCT | Australia | A single orthopaedic surgeon, private practice | Isolated ACL tear | >3 weeks but <12 months Mean: patellar - 19.8 ± 23 week; hamstring - 14.2 ± 15 week. | **Surgical:** 4-strand hamstring tendon ACL reconstruction | **Surgical:** Patellar tendon graft ACL reconstruction | 65 | 15y | No | No  (*post hoc*) |
| A20A Feller and Webster (2003) Am J Sports Med (3 years) | | | | | | | | | | | | | |

## Patellar Dislocation

|  | **Study (papers)** | **Journal** | **Design** | **Country** | **Setting** | **Condition** | **Time from injury to intervention/ enrolment** | **Intervention1** | **Intervention2** | ***N***  **[excl. after]** | **Study Time** | **1**°  **Out-come** | ***a priori* power calc.** |
| --- | --- | --- | --- | --- | --- | --- | --- | --- | --- | --- | --- | --- | --- |
| D1 | Apostolovic, Vukomanovic, Slavkovic, *et al.* (2011) | International Orthopaedics | Non-RCT. | Serbia |  | Acute patellar dislocation | Surgery ≤10 days; non-surgical immediately | **Surgical:** Arthroscopic surgery (standard procedure, for dislocation & other procedures as needed) | **Non-surgical:** Including closed reduction, immobilisation, cold packs, aspiration and exercises. | 37 | 6y | Yes | No |
| D2 | Bitar, Demange, D'Elia, *et al.* (2012) | Am J Sports Med | RCT | Brazil | Emergency department | Traumatic patellar dislocation | ≤3 weeks | **Surgical:** Medial patellofemoral ligament reconstruction | **Non-surgical:** Immobilization and physiotherapy | 39  (41 knees) | 2y | Yes | Yes  (*n*=22 per group) |
| D3 | Camanho, Viegas Ade, Bitar, *et al.* (2009) | Arthroscopy | RCT | Brazil |  | Acute patellar dislocation |  | **Surgical:** Medial patellofemoral ligament repair | **Non-surgical:** Immobilization followed by physiotherapy | 33 | 25m+ | Yes | Unclear |
| D4 | Christiansen, Jakobsen, Lund, *et al.* (2008). | Arthroscopy | RCT; randomized at point of arthroscopy | Denmark | Aarhus University Hospital | Primary patellar dislocation | Delayed arthroscopy: mean 50 days | **Surgical:** Initial treatment with brace then surgery *i.e.* delayed arthroscopy | **Non-surgical:** Initial treatment with brace then arthroscopy without repair | 80 | 2y | Yes | Yes  (*n*=39 per group) |
| D5 | Nikku, Nietosvaara, Aalto, *et al.* (2005) | Acta Orthop | RCT, 2 centres | Finland | 2 referral trauma centers | Primary patellar dislocation | ≤14 days | **Surgical:** Individually adjusted proximal realignment | **Non-surgical: I**mmobilisation (3 weeks) followed by functional rehabilitation | **127** | 7y | No | No |
| D5a Nikku, Nietosvaara, Kallio, *et al.* (1997) Acta Orthop Scand (2 years, ***n* = 125**) | | | | | | | | | | | | | |
| D6 | Palmu, Kallio, Donell, *et al.* (2008) | J Bone Joint Surg Am | RCT; children + adolescent | Finland | Hospital emergency department | Acute patellar dislocation | Within 2 weeks | **Surgical:** Direct repair of damaged medial structures if patella dislocatable, or lateral release alone if not + rehabilitation including orthosis | **Non-surgical:** Rehabilitation including orthosis | 62  (64 knees) | 14y | No | Yes  (*n*=16 per group) |
| D7 | Petri, Liodakis, Hofmeister, *et al.* (2013) | Arch Orthop Trauma Surg | RCT; multi-centre | Germany | Six German orthopaedic and trauma departments | Traumatic patellar dislocation |  | **Surgical:** Diagnostic arthroscopy followed by open soft tissue repairs + treatment as non-surgical arm | **Non-surgical:** Including brace with partial weight bearing with progression to full weight-bearing. | 24 | 2y | No | Yes  (*n*=65 per group) |
| D8 | Sillanpaa, Mattila, Maenpaa, *et al.* (2009) | J Bone Joint Surg Am | RCT; military recruits | Finland | Military hospital | Acute primary traumatic patellar dislocation |  | **Surgical:**  Initial surgical stabilization (1 of 2 initial stabilizing procedure) | **Non-surgical:** Orthosis | 40 | 7y | Yes | Yes  (*n*=16 per group) |

## Tibial plateau fracture

|  | **Study (papers)** | **Journal** | **Design** | **Country** | **Setting** | **Condition** | **Time from injury to intervention/ enrolment** | **Intervention1** | **Intervention2** | ***N***  **[excl after]** | **Study Time** | **1**°  **Out-come** | ***a priori* power calc.** |
| --- | --- | --- | --- | --- | --- | --- | --- | --- | --- | --- | --- | --- | --- |
| F1 | Bucholz, Carlton & Holmes (1989) [Abstract only] | Clin Orthop Relat Res | Non-RCT | USA |  | Tibial plateau fracture |  | **Surgical**: Cancellous autograft | **Surgical:** Interporous hydroxyapatite | 40? |  | - | Unclear |
| F2 | Canadian Orthopaedic Trauma Society (2006). | J Bone Joint Surg Am | RCT; multicentre; ITT principle | Canada | 5 university-affiliated level-I trauma centres | Displaced bicondylar tibial plateau fracture | Mean 3.7 +/-4.4 days (circular fixator); mean 4.2 +/- 4.6 days (open reduction) | **Surgical:** Standard open reduction and internal fixation with medial and lateral plates | **Surgical:** Percutaneous and/or limited open fixation and application of a circular fixator | 82  (83 Fx) | 2y | Yes | Yes  (*n*=40 per group) |
| F3 | Heikkila, Kukkonen, Aho, *et al.* (2011) | Journal of Materials Science: Materials in Medical | RCT | Finland | University Hospital -Orthopaedics | Depresssed unilateral tibial comminuted plateau fracture |  | **Surgical:** Bioglass (S53P4) | **Surgical:** Autogenous bone | 25 | 1y | No | No |
| F4 | Jiang, Luo, Wang, *et al.* (2008) | Knee | RCT | China | Team of surgeons | Bicondylar tibial plateau fractures |  | **Surgical:** Locked plate (Less Invasive Stabilization System, LISS) | **Surgical:** Classic double plates | 96  [12] | 2y+ | SS | Yes  (*n*=40 per group) |
| F5 | Jordan, Hao, Fader, *et al.* (2014). | Eur J Orthop Surg Traumatol | **PROTOCOL**  RCT; single centre | USA | Denver Health & Hospital Authority. 4 orthopaedic traumatologists | Depressed or split depressed tibial plateau fracture (medial or lateral) |  | **Surgical:** Balloon osteoplasty | **Surgical:** Traditional reduction techniques | 24 | 1y | Yes | No |
| F6 | Pernaa, Koski, Mattila, *et al.* (2011). | J Long Term Eff Med Implants | RCT follow up study | Finland | Department of Surgery, University Hospital | Tibial plateau fracture (joint-line depression >3mm) |  | **Surgical:** Bioactive glass (BAG)-S53P4 as bone-graft substitute | **Surgical:**  Autograft bone (AB) as bone-graft substitute | 29 | 11y | No | No |
| F7 | Russell, Leighton and Alpha (2008) [Abstract only] | J Bone Joint Surg Am | RCT; multicentre; 2:1 allocation |  |  | Acute, closed, unstable tibial plateau fractures |  | **Surgical:** Open reduction, standard plate-and-screw or screw-only fixation was used, then calcium phosphate cement for subarticular support. | **Surgical:** Open reduction, standard plate-and-screw or screw-only fixation was used,then bone graft subarticular support. | 119  (120 Fx) | 1y | No | Unclear |

## Other

|  | **Study (papers)** | **Journal** | **Design** | **Country** | **Setting** | **Condition** | **Time from injury to intervention/ enrolment** | **Intervention1** | **Intervention2** | ***N***  **[excl after]** | **Study Time** | **1**°  **Out-come** | ***a priori* power calc.** |
| --- | --- | --- | --- | --- | --- | --- | --- | --- | --- | --- | --- | --- | --- |
| O1 | Bailey, Minshull, Richardson, et al. (2014). | J Sport Rehabil | Pilot study; RCT [2:1 allocation] | UK | Orthopedic Hospital NHS Foundation Trust. Single consultant orthopaedic surgeon | Knee injury requiring Autologous chondrocyte implantation (ACI) | Mean 7.1 ± 4.9 mo (SD) | **Post-op rehab:**  Standardised non-concurrent physio-therapy [separation of strength and cardiovascular-endurance conditioning] | **Post-op rehab:** Standardised concurrent physiotherapy [strength & cardiovascular endurance conditioning] | 11 | 48w | Yes | No  (*post hoc*) |
| O2 | Ostojic, Arsic, Prodanovic, *et al.* (2007). | Res Sports Med | RCT | Serbia | Medical Centre, Out-Patient clinics | Acute knee injury | Within first 24 hours after injury | **Pharma:** Glucosamine (1500mg/day, 28d) | **Pharma:** Placebo | 121 | 28d | No | No |

1. Akelman MR, Fadale PD, Hulstyn MJ, Shalvoy RM, Garcia A, Chin KE, Duryea J, Badger GJ, Tung GA, Fleming BC. Effect of Matching or Overconstraining Knee Laxity During Anterior Cruciate Ligament Reconstruction on Knee Osteoarthritis and Clinical Outcomes: A Randomized Controlled Trial With 84-Month Follow-up. *Am J Sports Med* 2016; **44**(7): 1660-70.

2. Apostolovic M, Vukomanovic B, Slavkovic N, Vuckovic V, Vukcevic M, Djuricic G, Kocev N. Acute patellar dislocation in adolescents: operative versus nonoperative treatment. *Int Orthop* 2011; **35**(10): 1483-7.

3. Bailey AK, Minshull C, Richardson J, Gleeson NP. Improvement of outcomes with nonconcurrent strength and cardiovascular-endurance rehabilitation conditioning after ACI surgery to the knee. *J Sport Rehabil* 2014; **23**(3): 235-43.

4. Barenius B, Ponzer S, Shalabi A, Bujak R, Norlen L, Eriksson K. Increased risk of osteoarthritis after anterior cruciate ligament reconstruction: a 14-year follow-up study of a randomized controlled trial. *Am J Sports Med* 2014; **42**(5): 1049-57.

5. Beynnon BD, Johnson RJ, Naud S, Fleming BC, Abate JA, Brattbakk B, Nichols CE. Accelerated versus nonaccelerated rehabilitation after anterior cruciate ligament reconstruction: a prospective, randomized, double-blind investigation evaluating knee joint laxity using roentgen stereophotogrammetric analysis. *Am J Sports Med* 2011; **39**(12): 2536-48.

6. Beynnon BD, Uh BS, Johnson RJ, Abate JA, Nichols CE, Fleming BC, Poole AR, Roos H. Rehabilitation after anterior cruciate ligament reconstruction: a prospective, randomized, double-blind comparison of programs administered over 2 different time intervals. *Am J Sports Med* 2005; **33**(3): 347-59.

7. Bitar AC, Demange MK, D'Elia CO, Camanho GL. Traumatic patellar dislocation: nonoperative treatment compared with MPFL reconstruction using patellar tendon. *Am J Sports Med* 2012; **40**(1): 114-22.

8. Bucholz RW, Carlton A, Holmes R. Interporous hydroxyapatite as a bone graft substitute in tibial plateau fractures. *Clin Orthop Relat Res* 1989; (240): 53-62.

9. Camanho GL, Viegas Ade C, Bitar AC, Demange MK, Hernandez AJ. Conservative versus surgical treatment for repair of the medial patellofemoral ligament in acute dislocations of the patella. *Arthroscopy* 2009; **25**(6): 620-5.

10. Canadian Orthopaedic Trauma Society. Open reduction and internal fixation compared with circular fixator application for bicondylar tibial plateau fractures. Results of a multicenter, prospective, randomized clinical trial. *J Bone Joint Surg Am* 2006; **88**(12): 2613-23.

11. Christiansen SE, Jakobsen BW, Lund B, Lind M. Isolated repair of the medial patellofemoral ligament in primary dislocation of the patella: a prospective randomized study. *Arthroscopy* 2008; **24**(8): 881-7.

12. Cicuttini F, Wang Y, Marks P, Linklater J, Connell D, Hall S, Ghosh P, Skerrett D, Itescu S, Shimmin A. Randomized double-blind study of a single intraarticular injection of allogeneic mesenchymal precursor cells + hyaluronan compared to hyaluronan alone to explore clinical and structural outcomes in patients post anterior cruciate ligament reconstruction. *Osteoarthritis and Cartilage* 2016; **24**: S15.

13. Drogset JO, Grontvedt T, Robak OR, Molster A, Viset AT, Engebretsen L. A sixteen-year follow-up of three operative techniques for the treatment of acute ruptures of the anterior cruciate ligament. *J Bone Joint Surg Am* 2006; **88**(5): 944-52.

14. Frobell RB, Roos HP, Roos EM, Roemer FW, Ranstam J, Lohmander LS. Treatment for acute anterior cruciate ligament tear: five year outcome of randomised trial.[Reprint in Br J Sports Med. 2015 May;49(10):700; PMID: 25926596]. *Bmj* 2013; **346**: f232.

15. Heikkila JT, Kukkonen J, Aho AJ, Moisander S, Kyyronen T, Mattila K. Bioactive glass granules: a suitable bone substitute material in the operative treatment of depressed lateral tibial plateau fractures: a prospective, randomized 1 year follow-up study. *J Mater Sci Mater Med* 2011; **22**(4): 1073-80.

16. Jiang R, Luo CF, Wang MC, Yang TY, Zeng BF. A comparative study of Less Invasive Stabilization System (LISS) fixation and two-incision double plating for the treatment of bicondylar tibial plateau fractures. *The Knee* 2008; **15**(2): 139-43.

17. Jordan R, Hao J, Fader R, Gibula D, Mauffrey C. Study protocol: trial of inflation osteoplasty in the management of tibial plateau fractures. *Eur* 2014; **24**(5): 647-53.

18. Karimi-Mobarakeh M, Mardani-Kivi M, Mortazavi A, Saheb-Ekhtiari K, Hashemi-Motlagh K. Role of gracilis harvesting in four-strand hamstring tendon anterior cruciate ligament reconstruction: a double-blinded prospective randomized clinical trial. *Knee Surg Sports Traumatol Arthrosc* 2015; **23**(4): 1086-91.

19. Koken M, Akan B, Kaya A, Armangil M. Comparing the anatomic single-bundle versus the anatomic double-bundle for anterior cruciate ligament reconstruction: A prospective, randomized, single blind, clinical study. *European Orthopaedics and Traumatology* 2014; **5**(3): 247-52.

20. Kraus VB, Birmingham J, Stabler TV, Feng S, Taylor DC, Moorman CT, 3rd, Garrett WE, Toth AP. Effects of intraarticular IL1-Ra for acute anterior cruciate ligament knee injury: a randomized controlled pilot trial (NCT00332254). *Osteoarthritis Cartilage* 2012; **20**(4): 271-8.

21. Lawrence JT, Birmingham J, Toth AP. Emerging ideas: prevention of posttraumatic arthritis through interleukin-1 and tumor necrosis factor-alpha inhibition. *Clin Orthop* 2011; **469**(12): 3522-6.

22. Magnussen RA, Pottkotter K, Stasi SD, Paterno MV, Wordeman SC, Schmitt LC, Flanigan DC, Kaeding CC, Hewett TE. Femoral Nerve Block after Anterior Cruciate Ligament Reconstruction. *J Knee Surg* 2016; **30**: 30.

23. Meunier A, Odensten M, Good L. Long-term results after primary repair or non-surgical treatment of anterior cruciate ligament rupture: a randomized study with a 15-year follow-up. *Scand J Med Sci Sports* 2007; **17**(3): 230-7.

24. Nikku R, Nietosvaara Y, Aalto K, Kallio PE. Operative treatment of primary patellar dislocation does not improve medium-term outcome: A 7-year follow-up report and risk analysis of 127 randomized patients. *Acta Orthop* 2005; **76**(5): 699-704.

25. Ostojic SM, Arsic M, Prodanovic S, Vukovic J, Zlatanovic M. Glucosamine administration in athletes: Effects on recovery of acute knee injury. *Research in Sports Medicine* 2007; **15**(2): 113-24.

26. Palmu S, Kallio PE, Donell ST, Helenius I, Nietosvaara Y. Acute patellar dislocation in children and adolescents: a randomized clinical trial. *J Bone Joint Surg Am* 2008; **90**(3): 463-70.

27. Pernaa K, Koski I, Mattila K, Gullichsen E, Heikkila J, Aho A, Lindfors N. Bioactive glass S53P4 and autograft bone in treatment of depressed tibial plateau fractures - a prospective randomized 11-year follow-up. *J Long Term Eff Med Implants* 2011; **21**(2): 139-48.

28. Petri M, Liodakis E, Hofmeister M, Despang FJ, Maier M, Balcarek P, Voigt C, Haasper C, Zeichen J, Stengel D, Krettek C, Frosch KH, Lill H, Jagodzinski M. Operative vs conservative treatment of traumatic patellar dislocation: results of a prospective randomized controlled clinical trial. *Arch Orthop Trauma Surg* 2013; **133**(2): 209-13.

29. Russell TA, Leighton RK, Alpha BSMTPFSG. Comparison of autogenous bone graft and endothermic calcium phosphate cement for defect augmentation in tibial plateau fractures. A multicenter, prospective, randomized study. *J Bone Joint Surg Am* 2008; **90**(10): 2057-61.

30. Sillanpaa PJ, Mattila VM, Maenpaa H, Kiuru M, Visuri T, Pihlajamaki H. Treatment with and without initial stabilizing surgery for primary traumatic patellar dislocation. A prospective randomized study. *J Bone Joint Surg Am* 2009; **91**(2): 263-73.

31. Sun K, Tian SQ, Zhang JH, Xia CS, Zhang CL, Yu TB. Anterior cruciate ligament reconstruction with bone-patellar tendon-bone autograft versus allograft. *Arthroscopy* 2009; **25**(7): 750-9.

32. Sun K, Zhang J, Wang Y, Zhang C, Xia C, Yu T, Tian S. A prospective randomized comparison of irradiated and non-irradiated hamstring tendon allograft for ACL reconstruction. *Knee Surg Sports Traumatol Arthrosc* 2012; **20**(1): 187-94.

33. Swirtun LR, Jansson A, Renstrom P. The effects of a functional knee brace during early treatment of patients with a nonoperated acute anterior cruciate ligament tear: a prospective randomized study. *Clin J Sport Med* 2005; **15**(5): 299-304.

34. Tagesson S, Oberg B, Good L, Kvist J. A comprehensive rehabilitation program with quadriceps strengthening in closed versus open kinetic chain exercise in patients with anterior cruciate ligament deficiency: a randomized clinical trial evaluating dynamic tibial translation and muscle function. *Am J Sports Med* 2008; **36**(2): 298-307.

35. Taylor DC, DeBerardino TM, Nelson BJ, Duffey M, Tenuta J, Stoneman PD, Sturdivant RX, Mountcastle S. Patellar tendon versus hamstring tendon autografts for anterior cruciate ligament reconstruction: a randomized controlled trial using similar femoral and tibial fixation methods. *Am J Sports Med* 2009; **37**(10): 1946-57.

36. Thomee P, Wahrborg P, Borjesson M, Thomee R, Eriksson BI, Karlsson J. A randomized, controlled study of a rehabilitation model to improve knee-function self-efficacy with ACL injury. *J Sport Rehabil* 2010; **19**(2): 200-13.

37. Webster KE, Feller JA, Hartnett N, Leigh WB, Richmond AK. Comparison of Patellar Tendon and Hamstring Tendon Anterior Cruciate Ligament Reconstruction: A 15-Year Follow-up of a Randomized Controlled Trial. *Am J Sports Med* 2016; **44**(1): 83-90.

### Supplementary Table 3: Overview of inclusion/exclusion criteria categorised according to type of knee injury*.

* excludes conference abstracts and articles for which no full text could be obtained

|  | **Number of studies** | | | | |
| --- | --- | --- | --- | --- | --- |
|  | **ACL** | **Patellar Dislocation** | **Tibial plateau fracture** | **Other** | **Total** |
| Nos full papers/trials | **18**/20 | **7**/8 | **5**/7 | **2**/2 | **32**/37 |
| **Criteria clearly defined** | 16 | 7 | 4 | 1 | **28** |
| **Age range given** | 12 | 5 + 1x"young adult" | 2 |  | **19** |
| **Minimum age (years)** |  |  |  |  |  |
| missing |  |  | 1 |  | **1** |
| <12 |  | 1 |  |  | **1** |
| 12 - <16 | 4 | 4 |  |  | **8** |
| 16 - <18 | 2 |  |  |  | **2** |
| ≥18 | 6 |  | 1 |  | **7** |
| **Maximum age (years)** |  |  |  |  |  |
| missing |  | 1 |  |  | **1** |
| ≤20 |  | 2 (both ≤ 16) |  |  | **2** |
| >20-30 |  | 1 |  |  | **1** |
| >30 - 40 | 3 | 1 |  |  | **4** |
| >40 - 50 | 9 |  |  |  | **9** |
| >50 |  |  | 2 |  | **2** |
| **Sex** | 1 x men & women | 1 x men & women |  | 1 x men only | **3 specified** |
| **Pregnancy [exclusion]** | 4 | 1 (incl. lactating) | 1 |  | **6** |
| **Activity *e.g.* Tegner score** | 4 |  |  |  | **4** |
| **Sports activity level** | 1 |  |  | 1 | **2** |
| **Walking impairment, prior to injury [exclusion]** |  |  | 1 | 1 | **2** |
| **Previous injury [exclusion]** | 10 | 7 | 1 |  | **18** |
| both knees | 5 (incl. ACL or PCL tear) | 5 (incl. traumatic or recurrent dislocations, patellofemoral instability, disorder or pain) | 1 x fracture |  | **11** |
| index knee | 4 | 2 (incl. dislocation, subluxation, ligamentous injury, fracture, serious lesion or symptoms of patellofemoral instability) |  |  | **6** |
| lower extremities | 1 |  |  |  | **1** |

continued…

….continued

|  | **Number of studies** | | | | | |
| --- | --- | --- | --- | --- | --- | --- |
|  | **ACL** | **Patellar Dislocation** | **Tibial plateau fracture** | **Other** | | **Total** |
| **Previous surgery [exclusion]** | **12** | **4** |  |  | | **16** |
| both knees | 5 (incl. ACL reconstruction) | 3 |  |  | | **8** |
| involved knee | 6 (incl. cruciate ligament reconstruction) | 1 |  |  | | **7** |
| lower extremities | 1 |  |  |  | | **1** |
| **Osteoarthritis [exclusion]** | 6 (incl. radiographic; non-obese to minimize risk of pre-existing OA) |  | 1 |  | | **7** |
| **Pre-existing conditions [exclusion]** Including: | **6** | **1** | **4** | **1** | | **12** |
|  | 1. Systemic diseases including diabetes, rheumatic disease, cancer, neurological condition 2. Abnormal bone structure or diseases that predispose to articular cartilage damage 3. Symptomatic hip and/or ankle 4. History of DVT or coagulation disorders 5. Claustrophobia 6. Other injury including severe head injury (GCS <8), back pain that would negatively affect rehabilitation 7. Other conditions or treatment interfering trial completion, including patients with metal devices or movement disorders 8. Systemic medication/abuse of steroids 9. Currently taking, or previously treated with, glucosamine (or similar preparation) 10. Medical contra-indication to surgery 11. Requiring intensive care or requiring transfer to other departments for treatment | | | | | |
| **Concomitant contralateral knee injury/condition [exclusion]** | 5 (incl. injury to lower extremity [except partial knee meniscectomy]; ACL deficiency or reconstruction) | 1 (pathological condition) |  |  | **6** | |
| **Concomitant involved knee injury/condition [exclusion]** | **15** | **5** | **2** | **1** | **23** | |
|  | - See Table 2a | - [large >15mm] osteochondral fragments x2 - Serious/large osteochondral lesion [requiring surgery] x2 - Associated injuries - Non-traumatic patellar dislocation - Open injury - Anatomical deformities - Pre-existing pathology | - Pathological fracture x2 - Severe open fracture (Gustilo IIIB & IIIC) x2 - Ipsilateral femoral fracture - Open growth plates | - Injury >grade II |  | |

### Supplementary Table 4: Exclusion criteria for concomitant injury or condition of the involved (index) knee in ACL trials. One trial stipulated each criterion unless otherwise indicated. MCL-medial collateral ligament; LCL-lateral collateral ligament; PCL-posterior cruciate ligament; ROM-range of motion

| **Exclusion criteria given across 15 trials** | |
| --- | --- |
| Ligament injury   - Associated ligament injury - Multiple ligamentous injuries **x3** (incl. requiring surgery x1) - Lateral/posterolateral ligament complex injury with increased laxity - Increased clinical laxity of MCL, LCL or PCL   Collateral ligament   - Total collateral ligament rupture - Collateral ligament injury > grade II - A total rupture of MCL/LCL as visualized on MRI. - LCL injury **x2** - LCL rupture - MCL tear grade II or III **x3** (incl. requiring brace x1) - MCL injury grade III   Anterior cruciate ligament   - Partial ACL rupture **x2**   Posterior cruciate ligament   - PCL rupture - PCL insufficiency   PCL or posterolateral corner   - Associated PCL or posterolateral corner injuries **x5**   Fracture   - Simultaneous fracture **x3** | Meniscus   - Meniscal pathology - Associated meniscus injury - Suspected meniscal tear - Meniscal tear requiring repair **x3** (incl. where postop treatment i.e. bracing/limited ROM interferes with rehabilitation protocol x1) - Meniscal tears involving > ⅓ of meniscus - Bi-compartmental extensive meniscus resections   Articular cartilage/chondral lesions   - Chondral lesions - Moderate fissures or lesion in articular cartilage - Full-thickness cartilage/chondral lesion **x2** - Articular cartilage lesions with exposed bone **x3** - Articular cartilage damage ≥ grade III Outerbridge classification - Chondral lesions of a severity > Noyes grade IIA or Noyes grade IIA lesions > 1 cm in diameter - Chondral lesions grade III or IV by International Cartilage Repair Society criteria   Other   - Mal-alignment **x3** - Revision reconstruction **x2** - Septic joint - Radiographic: abnormal knee or evidence of chronic joint disease **x2** - history of intraarticular corticosteroid - Additional injury to lower extremity [except partial meniscal injury, minor collateral ligament injury or partial meniscectomy] |

### Supplementary Table 5: Physical examination outcomes used in acute knee injury studies. Abbreviations: SR – self-reported outcome measure; SS – based on use in sample size calculation; *n* Primary – used as primary outcome in *n* studies; +1 one study with both objective and SR measures.

| **Outcome measures (*n* = 30)** | **Number of studies** | | | | |
| --- | --- | --- | --- | --- | --- |
|  | **ACL**  ***n* = 20** | **Patellar Dislocation**  ***n* = 8** | **Tibial plateau fracture**  ***n* = 7** | **Other**  ***n* = 2** | **Total**  ***n* = 37** |
| Knee laxity (incl. knee (in)stability, instrumented) | 10 + 4 Primary (1 SR; 1 SS) |  | 2 (+1 SR) |  | 16 (4 Primary) |
| Generalized ligamentous laxity |  | 1 |  |  | 1 |
| Patellofemoral stability (incl. dislocation, subluxation) |  | 6 (+1 SR) + 2 Primary |  |  | 8 (2 Primary) |
| Collateral stability |  |  | 1 |  | 1 |
| Post treatment contralateral dislocations |  | 1 |  |  |  |
| Palpation (incl. effusion & crepitus) | 3 | 1 | 1 | 1 | 6 |
| Range of motion | 10 | 2 | 4 | 1 | 17 |
| Proprioception | 1 |  |  |  | 1 |
| Sensory change | 1 + 1 SR |  |  |  | 2 |
| Girth (knee and/or thigh) | 1 | 2 | 2 |  | 5 |
| Limb symmetry indices (QF-LSI). | 1 Primary (SS) |  |  |  | 1 Primary |
| Q-angle Knee alignment |  | 1 |  |  | 1 |
| Trochanter prominence angle test |  | 1 |  |  | 1 |
| Forced thigh foot angle |  | 1 |  |  | 1 |
| Anterior tibial displacement | 1 |  |  |  | 1 |
| Heel height | 1 |  |  |  | 1 |
| Patellar apprehension test |  | 1 |  |  | 1 |
| Gait testing - instrumented | 1 |  |  |  | 1 |
| McMurray test |  |  | 1 |  | 1 |
| Strength (incl. quadriceps and/or hamstrings) | 6 |  |  |  | 6 |
| Force (incl. peak force, rate of dev. & force error) |  |  |  | 1 Primary | 1 Primary |
| Torque | 4 + 1 Primary (SS) |  |  |  | 5 (1 Primary) |
| Muscle electrical activity | 1 |  |  | 1 Primary | 2 (1 Primary) |
| Functional - Jump | 3 |  | 2 |  | 5 |
| Functional – Squat | 1 |  | 2 |  | 3 |
| Functional – Hop | 8 | 1 |  | 1 Primary | 10 (1 Primary) |
| Functional – Walk | 2 (1 SR) |  | 2 (1 SR) |  | 4 |
| Functional - Stair climb | 1 SR |  | 2 (1 SR) |  | 3 |
| Function/Performance: running figure-of-8 | 1 | 1 |  |  | 2 |
| Functional recovery tools | 1 |  |  |  | 1 |

### Supplementary Table 6: Patient reported outcomes used in acute knee injury studies. Abbreviations: SR – self-reported outcome measure; *n* Primary – used as primary outcome in *n* studies; SS – included based on use in sample size calculation; OA - surrogate osteoarthritis outcome

| **Outcome measure** | **Number of studies** | | | | |
| --- | --- | --- | --- | --- | --- |
|  | **ACL**  ***n* = 20** | **Patellar Dislocation**  ***n* = 8** | **Tibial plateau fracture**  ***n* = 7** | **Other**  ***n* = 2** | **Total**  ***n* = 37** |
| Pain (incl. at rest, kneeling, walking, Werner score & provocation test) | 6 | 2 | 1 | 1 | 10 |
| Discomfort | 1 |  |  |  | 1 |
| Activity - self reported (incl. return to pre-injury levels) | 2 | 1 | 1 | 1 | 5 |
| Function - self reported (incl. SANE) | 3 |  | 1 |  | 4 |
| Satisfaction (incl. with intervention & activity levels) | 2 | 2 |  |  | 4 |
| Kujala score |  | 5 + 2 Primary |  |  | 7 (2 Primary) |
| Hospital for Special Surgery (HSS) knee score |  |  | 2 Primary (1 SS) |  | 2 Primary |
| Oxford Knee Score (OKS) |  |  | 1 |  | 1 |
| International Knee Documentation Committee (IKDC) Knee Form | 9 + 1 Primary (SS) |  |  | 1 Primary | 11 (2 Primary) |
| Knee injury Osteoarthritis Outcome Score (KOOS) | 11 + 4 Primary (1 SS) | 1 | 1 [**OA**] | 1 Primary | 18 (5 Primary) |
| The Knee Self-Efficacy Scale (K-SES) | 1 Primary |  |  |  | 1 Primary |
| Cincinnati knee score (incl. modified) | 4 |  |  |  | 4 |
| Lysholm Knee Scoring Scale (incl. modified & Lysholm II) | 8 | 1 |  |  | 9 |
| Hughston Visual Analogue Scale knee score |  | 2 |  |  | 2 |
| The Physical Activity Scale | 1 Primary |  |  |  | 1 Primary |
| Tegner activity score | 12 + 1 Primary | 3 |  |  | 16 (1 Primary) |
| Marx activity rating scale | 1 |  |  |  | 1 |
| Western Ontario and McMaster Universities Osteoarthritis Index (WOMAC) |  |  | 1 |  | 1 |
| Short Form-36 (SF-36) General Health Survey (incl. specific components, v2) | 4 |  | 1 |  | 5 |
| SF-12 |  |  | 1 |  | 1 |
| Veterans RAND 12-Item Health Survey (VR-12) | 1 |  |  |  | 1 |
| Multidimensional Health Locus of Control | 1 Primary |  |  |  | 1 Primary |
| Brace evaluation form | 1 |  |  |  | 1 |
| Treatment effect (incl. long term results) | 1 |  | 1 |  | 2 |
| Confidence in knee joint (VAS) | 1 |  |  |  | 1 |
| Fear of re-injury | 1 |  |  |  | 1 |

### Supplementary Table 7: Imaging outcomes used in acute knee injury studies. Abbreviations: *n* P – used as primary outcome in *n* studies; OA - osteoarthritis outcome; MFTC – medial femoro-tibial cartilage; Other – used in ‘other’ studies

| **Outcome measure** | **Number of studies** | | | |
| --- | --- | --- | --- | --- |
|  | **ACL** | **Patellar Dislocation** | **Tibial plateau fracture** | **Total** |
| **Radiographic** |  |  |  |  |
| - Sulcus angle |  | 3 |  | 3 |
| - Lateral patellofemoral angle |  | 2 |  | 2 |
| - Patellofemoral displacement (>1mm) |  | 1 |  | 1 |
| - Salvati index |  | 1 |  | 1 |
| - Insall-Salvati index |  | 1 |  | 1 |
| - Patella height |  | 2 |  | 2 |
| - Lateral patellar displacement |  | 1 |  | 1 |
| - Maturation of tibial apophysis |  | 1 |  | 1 |
| - Medial avulsion fracture of the patella |  | 1 |  | 1 |
| - Wiberg classification (patella shape) |  | 1 |  | 1 |
| - Presence of loose body |  | 1 |  | 1 |
| - Femoral tunnel widening | 1 |  |  | 1 |
| - Tunnel placement | 2 |  |  | 2 |
| - Articular subsidence |  |  | 1 | 1 |
| - Union rates |  |  | 1 | 1 |
| - Time to union |  |  | 1 | 1 |
| - Quality of reduction |  |  | 2 | 2 |
| - ***Radiographic OA*** | 9 + 1 P | 1 | 2 | 12 + 1 P |
| - *Medial joint space width* | *1* |  |  | *1* |
| - *Study specified criteria incl. joint space narrowing, osteophyte grade, subchondral sclerosis and sharpening of tibial spines.* | *2* |  | *2* | *4* |
| - *modified OARSI grading scale for OA* | *1* |  |  | *1* |
| - *Kellgren-Lawrence classification* | *3 + 1 P* |  |  | *3 + 1 P* |
| - *Ahlback & Fairbank composite scale* | *1* |  |  | *1* |
| - *Ahlback classification* | *1* | *1* |  | *2* |

| **Outcome measure** | **Number of studies** | | | |
| --- | --- | --- | --- | --- |
|  | **ACL** | **Patellar Dislocation** | **Tibial plateau fracture** | **Total** |
| **Computerised tomography (CT)** |  |  |  |  |
| - Cortical thickness (3cm below articular surface) |  |  | 1 | 1 |
| - Quality of reduction |  |  | 1 P | 1 P |
| **Radiologic (X-ray &/or CT)** |  |  |  |  |
| - Tibio-femoral angle |  |  | 2 | 2 |
| - Joint line depression |  |  | 1 | 1 |
| - Articular surface depression |  |  | 1 | 1 |
| - Morphological changes of bioglass granuales |  |  | 1 | 1 |
| - Deviation of mechanical axes (DMA) |  |  | 2 | 2 |
| **Magnetic resonance imaging (MRI)** |  |  |  |  |
| - Whole Organ Magnetic Resonance Imaging Score | 1 (OA) |  |  | 1 (OA) |
| - Anterior Cruciate Ligament Osteoarthritis Score | 1 P (OA) |  |  | 1 P (OA) |
| - Subregional changes in cartilage thickness | 1 |  |  | 1 |
| - Joint structural changes | 1 |  |  | 1 |
| - Articular cartilage lesions of patella facets & femoral condyles - numerical grading system, International Cartilage Repair Society (ICRS) |  | 1 |  | 1 |
| - Medial and lateral femur, tibia and patella bone surface areas of the knee | 1 |  |  | 1 |
| - Cartilage thickness of femorotibial cartilages: medial (MFTC) and lateral compartments | 1 P (MFTC) |  |  | 1 P (MFTC) |
| - Morphologic measures of articulating bone curvature (femur, tibia and trochlea) | 1 P |  |  | 1 P |
| - Fracture type | 1 |  |  | 1 |
| - Meniscal injuries (grade) | 1 |  |  | 1 |
| - Modifled Outerbridge classification |  |  |  | 1 other |
| **qMRI** |  |  |  |  |
| - Bone marrow lesions (position, volume) | 1 |  |  | 1 |
| - Early matrix changes typical of arthritis | 1 (OA) |  |  | 1 (OA) |

Supplementary Table 8: Biomarkers used as outcomes in acute knee injury studies. Biomarkers categorised by type, with source(s) i.e. synovial fluid (SF), serum or urine, and the number of studies used in. Abbreviations: Primary – used as a primary outcome measure.

| **Biomarker** | **Source** | **Number ACL studies** |
| --- | --- | --- |
| **Inflammation** |  |  |
| IL-1α | SF &/or serum | 2 |
| IL-1β | SF &/or serum | 2 |
| IL-1Ra | SF &/or serum | 2 |
| IL-6 | SF &/or serum | 1 |
| IL-8 | SF &/or serum | 1 |
| IL-10 | SF &/or serum | 1 |
| IFNγ | SF &/or serum | 1 |
| TNFα | SF &/or serum | 2 |
| hsCRP | SF | 1 |
| sCD44 - other | SF &/or serum | 1 |
| **Matrix components** |  |  |
| CILP (Cartilage Intermediate Layer Protein) | Serum | 1 |
| Aggrecan (846 epitope) | SF | 1 |
| Hyaluronan | SF &/or serum | 2 |
| Tenascin C | SF &/or serum | 1 |
| Cartilage oligomeric matrix protein (COMP) | SF &/or serum | 2 |
| Lubricin | SF &/or serum | 1 |
| **Collagen metabolism** |  |  |
| C1,2C | SF &/or serum | 1 |
| C2C | SF &/or serum | 1 |
| COL2-3/4CLong neoepitope | SF | 1 |
| CPII | SF &/or serum | 2 |
| CTxI | SF &/or serum | 1 |
| CTxII | SF &/or serum or urine | 2 |
| NTX | SF &/or serum | 1 |
| NTX-I (N-terminal type I) | Urine | 1 |
| Osteocalcin | SF &/or serum | 1 |
| **Biomarker** | **Source** | **Number ACL studies** |
| **GAG/proteoglycan metabolism** |  |  |
| ARGS-aggrecan | SF &/or serum | 1 + 1 Primary |
| CS846 | SF &/or serum | 1 |
| Fetal aggrecan FA846 | SF &/or serum | 1 |
| GAG (Glycosaminoglycan) | SF | 1 |
| sGAG (sulphated Glycosaminoglycan) | SF &/or serum | 1 |
| **Post-translational markers of protein age** |  |  |
| β-Aspartate | SF &/or serum | 1 |
| D-Asp/protein | SF | 1 |
| D-Asx | SF &/or serum | 1 |
| D-Serine | SF &/or serum | 1 |
| PIMT/protein | SF | 1 |
| **Proteinases/other** |  |  |
| Cathespsin K | SF | 1 |
| Total protein | SF | 1 |
| MMP-3 | SF &/or serum | 1 |
| Immunogenicity (Anti-Human leukocyte antigen (HLA) panel reactive antibodies (PRA) against Class I & II HLAs) |  | 1 |

### Supplementary Table 9: Other outcomes used in acute knee injury studies. Abbreviations: Primary – used as a primary outcome measure.

| **Outcome measure** | **Number of studies** | | | | |
| --- | --- | --- | --- | --- | --- |
|  | **ACL**  ***n* = 20** | **Patellar Dislocation**  ***n* = 8** | **Tibial plateau fracture**  ***n* = 7** | **Other**  ***n* = 2** | **Total**  ***n* = 37** |
| Work (capacity, and return to work) |  |  | 2 |  | 2 |
| Complications post-op incl. sequelae of treatment | 1 | 1 | 3 |  | 5 |
| Further operations | 1 | 2 | 3 |  | 6 |
| Time in rehabilitation with physical therapist | 1 |  |  |  | 1 |
| Fracture healing time |  |  | 1 |  | 1 |
| Length of hospital stay post surgery |  |  | 2 |  | 2 |
| Need for supporting device |  |  | 1 |  | 1 |
| Safety, tolerability & adverse events (unspecified) | 1 Primary |  |  |  | 1 Primary |
| Roentgenographic and clinical assessments (unspecified) |  |  | 1 |  | 1 |
